# Endogenous Morphine Levels Are Increased in Sepsis: A Partial Implication of Neutrophils

**DOI:** 10.1371/journal.pone.0008791

**Published:** 2010-01-20

**Authors:** Elise Glattard, Ingeborg D. Welters, Thomas Lavaux, Arnaud H. Muller, Alexis Laux, Dan Zhang, Alexander R. Schmidt, François Delalande, Benoît-Joseph Laventie, Sylvie Dirrig-Grosch, Didier A. Colin, Alain Van Dorsselaer, Dominique Aunis, Marie-Hélène Metz-Boutigue, Francis Schneider, Yannick Goumon

**Affiliations:** 1 Inserm, U575, Physiopathologie du Système Nerveux and Nociception and Pain Department, Institut des Neurosciences Cellulaires et Intégratives, Centre National de la Recherche Scientifique, Université de Strasbourg, Strasbourg, France; 2 Critical Care Research Unit, School of Clinical Sciences, University of Liverpool, Liverpool, United Kingdom; 3 Service de Réanimation Médicale, Hôpitaux Universitaires et Faculté de Médecine, Université de Strasbourg, Hôpital de Hautepierre, Strasbourg, France; 4 Laboratoire de Physiopathologie des Interactions Hôte-Bactérie, EA3432, Institut de Bactériologie, Université de Strasbourg, Strasbourg, France; 5 Abteilung für Anaesthesiologie, Intensivmedizin und Schmerztherapie, Universitätsklinikum Giessen und Marburg, Standort Giessen, Giessen, Germany; 6 Laboratoire de spectrométrie de masse BioOrganique, IPHC-DSA, ULP, CNRS UMR7178, Strasbourg, France; University of Giessen Lung Center, Germany

## Abstract

**Background:**

Mammalian cells synthesize morphine and the respective biosynthetic pathway has been elucidated. Human neutrophils release this alkaloid into the media after exposure to morphine precursors. However, the exact role of endogenous morphine in inflammatory processes remains unclear. We postulate that morphine is released during infection and can be determined in the serum of patients with severe infection such as sepsis.

**Methodology:**

The presence and subcellular immunolocalization of endogenous morphine was investigated by ELISA, mass spectrometry analysis and laser confocal microscopy. Neutrophils were activated with Interleukin-8 (IL-8) or lipopolysaccharide (LPS). Morphine secretion was determined by a morphine-specific ELISA. μ opioid receptor expression was assessed with flow cytometry. Serum morphine concentrations of septic patients were determined with a morphine-specific ELISA and morphine identity was confirmed in human neutrophils and serum of septic patients by mass spectrometry analysis. The effects of the concentration of morphine found in serum of septic patients on LPS-induced release of IL-8 by human neutrophils were tested.

**Principal Findings:**

We confirmed the presence of morphine in human neutrophil extracts and showed its colocalisation with lactoferrin within the secondary granules of neutrophils. Morphine secretion was quantified in the supernatant of activated human polymorphonuclear neutrophils in the presence and absence of Ca^2+^. LPS and IL-8 were able to induce a significant release of morphine only in presence of Ca^2+^. LPS treatment increased μ opioid receptor expression on neutrophils. Low concentration of morphine (8 nM) significantly inhibited the release of IL-8 from neutrophils when coincubated with LPS. This effect was reversed by naloxone. Patients with sepsis, severe sepsis and septic shock had significant higher circulating morphine levels compared to patients with systemic inflammatory response syndrome and healthy controls. Mass spectrometry analysis showed that endogenous morphine from serum of patient with sepsis was identical to poppy-derived morphine.

**Conclusions:**

Our results indicate that morphine concentrations are increased significantly in the serum of patients with systemic infection and that morphine is, at least in part, secreted from neutrophils during sepsis. Morphine concentrations equivalent to those found in the serum of septic patients significantly inhibited LPS-induced IL-8 secretion in neutrophils.

## Introduction

Morphine was first identified in opium from *Papaver somniferum,* and is one of the strongest known analgesic compounds [1]. Endogenous morphine has been characterized in several mammalian cells and tissues [2], [3], [4]. In mammals, the biosynthesis of endogenous morphine is associated with dopamine [5], [6], [7], as demonstrated in the SH-SY5Y human neuronal catecholamine-producing cell line [8], [9]. More recently, we showed that μ opioid receptors as well as their ligands morphine and morphine-6-glucuronide (M6G) are present in the human neuroblastoma SH-SY5Y cell line and that morphine is secreted from the large dense core vesicles in response to nicotine stimulation *via* a Ca^2+^-dependent mechanism [10].

Endogenous morphine or precursors were also found in peripheral organs including adrenal gland [3], [11] and liver [12], [13]. In addition, our group reported the presence of morphine-6-glucuronide (M6G) bound to phosphatidylethanolamine-binding protein/RKIP [14], in the secretory granules and secreted material of bovine adrenal chromaffin cells [14], [15]. Secretion of endogenous alkaloids together with catecholamines into the blood is likely to occur during stress situations and could be involved in different stress- or pain-modulating mechanisms *via* binding to μ opioid receptors expressed on numerous cell types including endothelial and immune cells [16], [17], [18].

Recently, new insights were gained from studies showing the production of morphine by human polymorphonuclear cells (PMN) [19]. However, the presence of morphine in neutrophils as well as the occurrence of morphine in serum is matter of debate, in particular, since morphine production could be attributed to either erythrocytes [20] or neutrophils. Human neutrophils were shown to be able to release morphine into the media after exposure to precursors including L-tyrosine, L-DOPA, tetrahydropapaveroline (THP) and reticuline. Stimuli such as alcohol, nicotine, and cocaine induce morphine release from human white blood cells *in vitro* [21]. Furthermore, a nonclassical cholinergic regulation of morphine release from human white blood cells was demonstrated [22]. Leukocytes play an important role in innate immune responses and represent a major defense mechanism against infection. During sepsis, PMN are also involved in organ dysfunction, such as acute lung injury or acute kidney failure [23].

However, the role of endogenous morphine in inflammation remains unclear, and knowledge of its secretion from immunocytes as well as its subcellular localization is lacking. Several studies suggest a role of endogenous morphine in maintaining homeostasis as part of the response to stress, in particular inflammation or infection. Interestingly, a recent paper reported that low concentrations of morphine (10–100 nM) enhanced migration of primary microglial cells toward adenosine diphosphate *via* a μ opioid receptor-dependant mechanism [24].

Endogenous morphine levels have been investigated after surgical intervention and have been found to be elevated after cardiovascular bypass [25], [26]. Morphine concentrations in the blood are higher after open cholecystectomy compared with laparoscopic cholecystectomy as the less invasive surgical procedure [27]. In addition, LPS administration to rats dramatically increased the amount of circulating and cerebral endogenous morphine [28], [29]. An increase of morphine in blood was also observed upon fasting conditions [12].

The present study focuses on the presence of endogenous morphine in the serum of patients with sepsis. First, we have shown the presence of morphine in secondary granules containing lactoferrin and characterized the secretion of morphine as well as expression of the μ opioid receptor in LPS- and IL-8 stimulated human neutrophils from healthy donors. Effects of low concentration of morphine on LPS-induced IL-8 secretion from human neutrophils were also studied. Secondly, we determined morphine levels in the serum of septic patients. Together, our data suggest that in patients with systemic inflammation and infection morphine secretion into the blood is part of their immune response and might represent a biological marker of these conditions.

## Materials and Methods

### Isolation of Human PMN

Human PMN were prepared from buffy coats obtained from EFS (Etablissement Français du Sang, Strasbourg, France) by centrifugation as previously described [30]. Briefly, 50 ml of buffy coat were diluted (2∶3, v∶v) in sodium chloride buffer (0.9%, w∶v). A 30 ml suspension was gently loaded on 12 ml of lymphocyte separation medium (Eurobio, France). After centrifugation (800 g, 20 min), pellets containing PMN were washed with NaCl 0.9%. PMN were suspended in 30 ml NaCl 0.9% and 10 ml Dextran 6% (U.S.B., USA), and mixed vigorously. After 1 hour of sedimentation, the supernatant was recovered and centrifuged (800 g, 15 min). Pellets were washed in NaCl 0.9% and resuspended in erythrocyte lysis buffer (Qiagen, France), kept 5 min on ice before addition of NaCl 0.9%, then centrifuged (800 g, 15 min). Pellets of PMN were finally suspended in 10 ml RPMI 1640 (Sigma-Aldrich, France). The cell population obtained with this technique contains more than 95% neutrophils [31]. Cell viability and cell yield were evaluated by Trypan blue exclusion, using a Neubauer chamber and an optic microscope. For none of the *in vitro* experiments a significant increase in cell death compared to controls was observed.

### Immunohistochemistry

In order to observe the presence of morphine immunoreactivity in both neutrophils and erythrocytes, a drop of fresh healthy human blood was spread on a glass slide and let dry for 3 min at RT. Blood cells were fixed with 5 soaking of 1 sec in 100% methanol at RT. Cells were permeabilized with a solution of PFA 2% in acetone (v∶v) during 2 min at −20°C. After drying the immunodectection was carried on as described below.

Cytospins were prepared by centrifugation of 2.5.10^5^ isolated PMNs onto glass slides (800 g, 10 min). Cells were fixed 20 min with 4% paraformaldehyde (w∶v, Sigma Aldrich, France) in phosphate-buffered saline (PBS, 0.9% NaCl and 25 mM sodium phosphate, pH 7.4; Euromedex, France). Cells were permeabilized for 1 min with 0.05% (v∶v) Triton X100 in PBS [15].

Immunostaining was performed as previously described [32]. Slides were washed in PBS and incubated for 1 h in horse serum diluted in PBS (10%, v∶v) in order to saturate nonspecific immunoreactive sites. After six PBS washes (5 min), coverslips were incubated overnight in different antisera. Primary antibodies were used as follows: (*i*) mouse monoclonal 6D6 (Aviva System Biology, USA; dilution 1∶2000) raised against morphine-like compounds (morphine, morphine-3-glucuronide, and M6G, based on supplier specifications and our own experiments) and (*ii*) rabbit anti-lactoferrin polyclonal antibody (dilution 1∶5000) [33].

After incubation with the primary antibody, slides were washed six times with PBS (5 min) and specific secondary antisera were added for 2 h at room temperature, followed by six PBS washes (5 min). These secondary antisera were (*i*) Cy5-conjugated donkey anti-mouse IgG (Jackson Immunoresearch Laboratories, USA, dilution 1∶2,000) and (*ii*) Cy3-conjugated donkey anti-rabbit IgG (Jackson Immunoresearch Laboratories; dilution 1∶2000).

**Figure 1:**
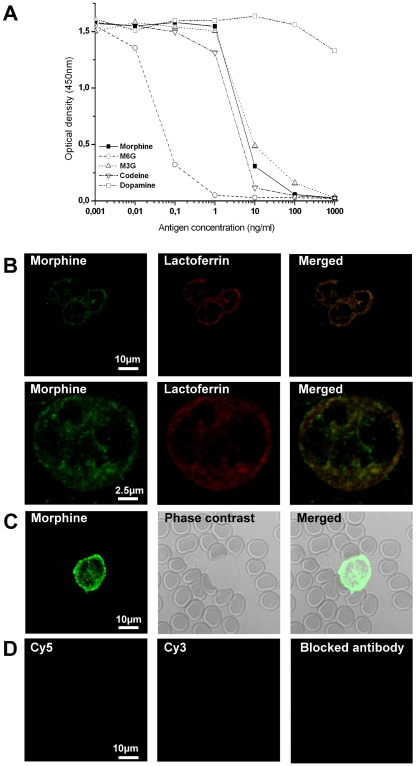
Evidence of the presence of morphine-like immunoreactivity in human neutrophils. ***A*** *. ELISA showing the specificity of the 6D6 antibody.* ***B*** *. Upper panel*, double immunofluorescence confocal micrographs performed on purified neutrophils from healthy donors. Labeling was performed with a mouse anti-morphine antibody (visualized in green pseudocolor with a Cy5-conjugated IgG) and with an antibody against lactoferrin (a secondary granule marker) visualized in red pseudocolor with a Cy3-conjugated IgG. Colocalized immunolabelling (merged window) appears as yellow staining. *Lower panel*, higher magnification. ***C.*** Immunofluorescence confocal micrographs performed on a blood drop from an healthy donor. Labeling was performed with a mouse anti-morphine antibody (visualized in green pseudocolor with a Cy5-conjugated IgG). Phase contrast allows to observe both neutrophils and erythrocytes. Merged window correspond to the supperposition of the two pictures. ***D.*** To assess the specificity of the secondary antibody, control experiments were performed using Cy3-conjugated IgG or Cy5-conjugated IgG without primary antibody. Controls for morphine immunoreactivity were carried out by preincubating the antibody with morphine.

Several controls were carried out to assess antibody specificity and nonspecific immunoreactivity. Primary antibodies were omitted, and each secondary antibody was tested individually or in a mixture in the presence of cells. Control for morphine antibody was carried out by incubating the antibody with morphine (2 h, 25°C, 50∶1, w/w), prior to immunocytochemistry experiments. Each antibody was also tested with the secondary antibody used for the second immunolabelling in order to determine whether interspecies cross-reactivity exists. Anti-morphine 6D6 antibody was also tested by ELISA in order to determine cross reactivity with morphine, M6G, and morphine-3-glucuronide (M3G), showing a specificity for morphine, M6G, M3G, and codeine (Fig. 1A). No cross reactivity was found for adrenaline, noradrenaline, dopamine and tetrahydropapaveroline (Sigma-Aldrich, France).

### Confocal Microscopy

Immunofluorescent staining was analyzed with a Leica laser scanning microscope (TCS-SP2 invert) equipped with a plan apo 63X oil immersion lens. Tissue sections were subjected to optical serial sectioning to produce images in the X–Y plane. Each optical section was scanned eight times for brain sections and four times for cells to obtain an average image. Pictures were recorded digitally in a 512×512 pixel format. A look-up table (glowoverglowunder, Leica) ensured that the full dynamic range of the photomultipliers was used. Before each measurement, a series of sections was acquired through the vertical axis in order to choose the equatorial section.

### Stimulation of PMN with LPS

Immediately after isolation, 20.10^6^ neutrophils in 200 µl of EGTA buffer (140 mM NaCl, 5 mM KCl, 10 mM glucose, 0.1 mM EGTA, 10 mM Hepes, 3 mM Tris; pH 7.3) with or without 1.1 mM CaCl_2_, were preincubated in low binding tubes for 30 min (37°C, 5% CO_2_) prior to stimulation. To stimulate PMN, LPS (*E. coli, O111:B4*; 10 ng/ml in 200 µl reaction volume, Sigma-Aldrich) was added to the medium and cells were incubated for 6 h at 37°C, 5% CO_2_ [34]. After incubation, supernatants were recovered by centrifugation (800 g, 10 min), and 40 µl was tested (in duplicate) for the presence of morphine using a morphine-specific ELISA kit (see below). In controls, LPS was replaced by EGTA buffer with and without Ca^2+^ Additional control experiments with LPS alone (without cells) revealed an absence of cross-reactivity with the ELISA. Secretion efficiency was checked by Western blot analysis (see below) using an antibody against a secretion marker (*i.e.,* lactoferrin).

### Stimulation of Neutrophils with IL-8

Neutrophils (20.10^6^ cells) were preincubated in 200 µl EGTA buffer with or without 1.1 mM CaCl_2_ for 30 min at 37°C prior to stimulation. Neutrophils were stimulated in 200 µl reaction volumes, as previously described [34], [35]. Cells were incubated with cytochalasin B (5 µg/ml, 5 min, Sigma-Aldrich) prior to IL-8 stimulation (50 nM, 7 min; R&D Systems Europe, France). After centrifugation (800 g, 10 min), supernatants were recovered and analyzed using a morphine-specific ELISA kit (see below). In controls, IL-8 was replaced by EGTA buffer with and without Ca^2+^. Additional control experiments with IL-8 and cytochalasin B alone (without cells) revealed an absence of cross-reactivity with the ELISA. Secretion efficiency was checked by Western blot analysis (see below) using an antibody against a secretion marker (*i.e.,* lactoferrin).

### Alkaloid Analysis

10 ml of serum from septic patients (n = 3), as well as serum from healthy donors (n = 3), human neutrophils (140 to 200.10^6^ cells) and alkaloid standards, were deproteinized as previously described [36]. Deproteinized sample extracts were dissolved in 0.1% trifluoroacetic acid in H_2_O (v∶v) prior to solid phase extraction. Samples were loaded on a Sep Pack cartridge (Waters Corporation) previously activated with 10 ml of 100% acetonitrile and washed with a solution of 0.1% trifluoroacetic acid in H_2_O (v∶v). Elution was performed with a solution of 15% acetonitrile with 0.1% trifluoroacetic acid in H_2_O (v∶v∶v). Eluted samples were dried with a SpeedVac evaporator. In order to determine the recovery of morphine present in the serum samples, 4 different amounts of exogenous morphine were added to 1 ml of morphine-free serum (0.5, 1, 5 and 10 ng/ml, n = 3). The percentage of recovery calculated for the extraction steps before the mass spectrometry (MS) analysis was 62.7+/−11%.

### Mass Spectrometry

Mass spectrometry (MS) and MS-MS analyses were performed using electrospray mass spectrometry on a Q-TOF II (quadrupole-time of flight, Bio-Tech) in positive mode as previously described [10], [15].

### Gel Electrophoresis and Western Blot Analysis

Proteins were separated on SDS-PAGE gradient gels (4%–12% acrylamide; Criterion XT, BioRad) and electrotransferred onto polyvinyldifluorene membranes (GE Healthcare Bioscience, Sweden) [37]. SH-SY5Y cell extract was used as a positive control. Human lactoferrin (75 kDa) was detected using a rabbit polyclonal antibody (dilution 1∶5,000 [33]) and revealed using HRP-conjugated anti-rabbit antisera (Sigma Aldrich, dilution 1∶400,000) and Supersignal West Femto Kit (Pierce, USA). For μ opioid receptor detection, 50 µg of neutrophil extract was loaded on the gel. The μ opioid receptor was detected using a goat anti-MOR1 antibody (N-20; Santa Cruz Biotechnology, USA; dilution 1∶3,000) and revealed using HRP-conjugated anti-goat antisera (Jackson immunoresearch, England; dilution 1∶50,000). Control experiments omitting the primary antibody confirmed the specificity of the label. Apparent molecular weights were evaluated by comparison with molecular weight standards (Bio-Rad).

### Morphine-Specific ELISA

**Figure 2:**
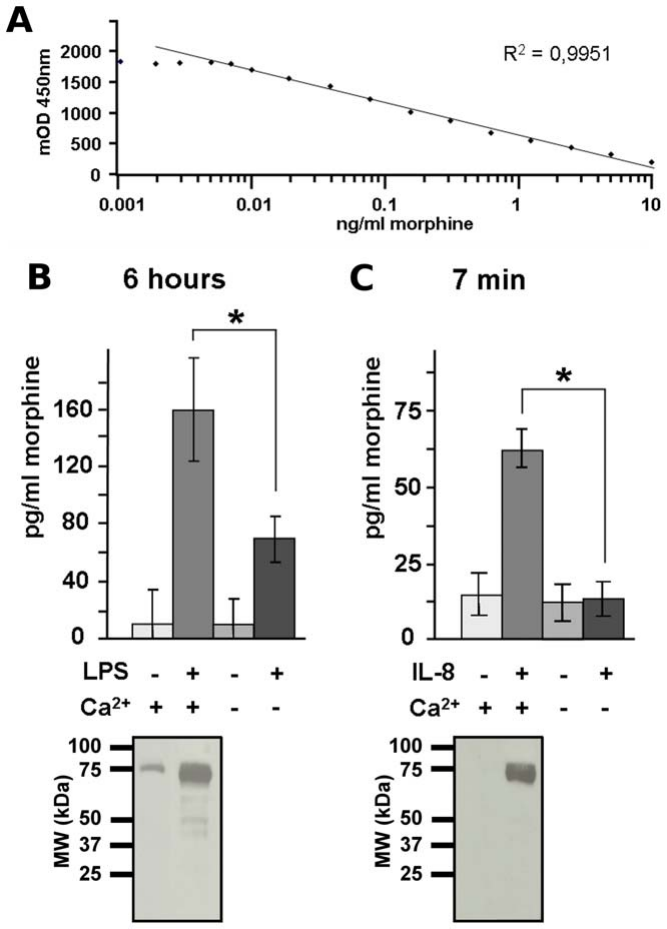
Morphine secretion from human primary neutrophils. ***A*** *.* Typical dose-response curve of the morphine detection performed with the morphine-specific ELISA. ***B. Upper panel***, Morphine secreted from neutrophils expressed as pg/ml. Morphine was quantified in culture medium after stimulating 20.10^6^ neutrophils with LPS (10 ng/ml, 6 h; n = 6) in presence or absence of Ca^2+^. Basal secretion levels were obtained from neutrophils incubated without LPS (6 h; n = 6). *Lower panel*, Efficiency of secretion was assessed by monitoring the secretion of lactoferrin (75 kDa, a marker of secondary granules) by Western blot analysis. ***C.*** ** ***Upper panel*** **,** Morphine was quantified in culture medium after stimulating 20.10^6^ neutrophils with IL-8 (50 nM, 7 min; n = 7) in presence or absence of Ca^2+^. Basal secretion levels were obtained from neutrophils incubated without IL-8 (7 min; n = 6). ***Lower panel***, The efficiency of secretion was assessed by monitoring the secretion of lactoferrin (75 kDa, a marker of secondary granules) by Western blot analysis (50 µg). Amounts of secreted morphine in the LPS and IL-8 groups were statistically different from the two control groups (no LPS and IL-8; *: p<0.01 with a Mann-Whitney test after Bonferroni correction.

A morphine-specific ELISA kit (Immunalysis Corporation, USA) was used for determination of morphine present in culture medium (40 µl in duplicate; n = 6 or 7) or in the serum of patients (20 µl, in duplicates). The specificity of the test for morphine was confirmed by testing different amounts of dopamine, adrenaline, noradrenaline, M6G, M3G, and codeine (0–25 ng/ml, data not shown). For all tests, morphine standards were diluted in the appropriate buffer. The CV values were between 0% to 8%. All samples with a higher CV value were retested in order to obtain a CV below or equal to 8%. The calculated methodological detection limit of the batch of ELISA kit use for the study was 0.01 ng/ml of morphine. No cross reactivity for LPS, IL-8, or cytochalasin B was found (*i.e*., the values of morphine were under the detection limit). The linearity of morphine detection in morphine-free serum is illustrated in Fig. 2A.

### IL-8 Secretion Assay and Quantification

Neutrophils were treated as previously described [38]. Briefly, neutrophils (1.10^6^ cells/well) were cultured in 24-well plates in 1 ml of complete RPMI 1640 nutrient (Sigma Aldrich) supplemented with 10 mM HEPES (Sigma Aldrich), 2 mM L-glutamine (Sigma Aldrich), 100 U/ml penicillin, 100 µg/ml streptomycin, 0.25 µg/ml amphotericin B, and10% fetal bovine serum in a moist atmosphere containing 5% CO_2_ at 37°C for 24 h in the presence of 10 ng/ml LPS, 8nM morphine (*i.e*., 2.25ng/ml) and/or 10 µM naloxone. LPS and morphine were added to cultures simultaneously, whereas the naloxone was added 30 min before treatments. Supernatants from cell cultures were collected, centrifuged for 10 min at 800 g, and stored in the frozen state at −20°C.

Quantification of IL-8 present in the medium of stimulated cells was assayed with a commercial human IL-8 ELISA (BD Biosciences, USA) according to manufacturer instructions. ELISA sensitivity for IL-8 was 0.8 pg/ml with a working range being between 0.8 to 200 pg/ml. If required, samples were diluted with the buffer used for the stimulation assay. The ELISA was performed on 100 µl of samples in duplicates. The CV values were between 0% and 8%. All samples with a higher CV value were retested in order to obtain a CV below or equal to 8%. No cross-reactivity for LPS, morphine or naloxone was found (data not shown).

### Validation of the 6D6 Antibody by ELISA

Morphine 6D6 antibody specificity was tested on a 96 wells plate (NUNC, Roskilde, Denmark) coated overnight at room temperature with 200 µL of a 100 ng/ml morphine-BSA solution (Fitzgerald Industries International, USA). After three washes with PBS-0,05% tween buffer, wells were incubated for 1 hour with 200 µl of BSA diluted in PBS buffer (5%, w/v) in order to saturate nonspecific sites. After saturation wells were incubated 30 min with 100 µl of PBS-BSA (3%, w/v) containing 0 ng/ml, 0.02 ng/ml, 0.2 ng/ml, 2 ng/ml, 20 ng/ml, 200 ng/ml and 2 µg/ml of the following potential antigens: morphine (Sigma-Aldrich), M6G (Sigma-Aldrich), M3G (Sigma-Aldrich), codeine (Sigma-Aldrich) and dopamine (Sigma-Aldrich). 100 µl of the mouse monoclonal primary antibody (6D6, Aviva System Biology; dilution 1∶2000) in PBS-BSA (3%, w/v) were added in each wells. After 3 hours incubation at RT, the plate was washed three times with PT buffer and 200 µl of the secondary antibody (HRP-conjugated donkey anti-mouse IgG, P.A.R.I.S.; dilution 1∶400) was added and let in the wells during 2 hours at RT. After two washes with PBS-0,05% tween buffer followed with two washes with a pH 7,5 phosphate-citrate-0,05% tween buffer (CT), revelation was performed with 200 µl of a freshly solution of ortho-penylene diamine (OPD; Sigma Aldrich) at 1,5 mg/ml in CT buffer containing 0,075% hydrogen peroxide. After 20 min of incubation at RT, optical density was determined at 450 nm on a Multiskan EX plate reader (Thermo Life Sciences, France). The 50% displacement point of the antibody for an antigen is determined with KyPlot 2.0 (after a sigmoidal fit of the results).

### Flow Cytometric Determination of μ Opioid Receptor Expression on Neutrophils

μ opioid receptor expression was determined as described before [39]. Briefly, 100 µl blood was stimulated with LPS (100 ng/ml) for 3, 6, 12 and 24 h (37°C in a 95% air/5% CO_2_ atmosphere). Incubation with NaCl 0.9% served as control. Samples were spun down (10 min, 250 g) and incubated with (FITC)-conjugated antibodies against the N-terminus (N-20; Santa Cruz Biotechnology) or the the C-terminus (C-20; Santa Cruz Biotechnology) of the μ opioid receptor for 15 min. FITC-labeled antibodies against human IgG were used to rule out unspecific binding and for cytometer setup. All antibodies were purchased with FITC tag (Camon-Serotech, Germany). Samples were spun down after incubation with anti-MOR (10 min, 250 g). For red cell lysis, Facs® Brand Lysing Solution (Becton Dickinson, Germany) was added. After incubation for 10 minutes samples were centrifuged (5 min, 250 g), washed twice with PBS and analyzed by flow cytometry. Flow cytometric analysis was performed using a FACSCalibur® (Becton Dickinson) flow cytometer with argon ion laser excitation at 488 nm, with 15000 events of each sample measured. Data were acquired and processed using CellQuest® software. Neutrophils were identified and live gated by their special characteristics in forward angle light scatter (FSC) and 90° side scatter (SSC). Medians of fluorescence intensities (MFI) of antibody-stained granulocytes were determined as a measure of μ opioid receptor expression. Using histogram analysis, the percentage of neutrophils which responded to stimulation with a high μ opioid receptor expression was determined.

### PCR and Gel Eletrophoresis

Neutrophils (5.10^6^ cells/well) were cultured in 24-well plates in 1 ml of complete RPMI 1640 nutrient (Sigma Aldrich) supplemented with 10 mM HEPES (Sigma Aldrich), 2 mM L-glutamine (Sigma Aldrich), 100 U/ml penicillin, 100 µg/ml streptomycin, 0.25 µg/ml amphotericin B, and10% fetal bovine serum in a moist atmosphere containing 5% CO2 at 37°C for 24 h in the presence of 100 ng/ml LPS. Total RNA was isolated using RNeasy columns (Quiagen, USA) according to the manufacturer's protocols. The cDNA synthesis reaction were performed with 1 µg of total mRNA and 100 U of SuperScript II reverse transcriptase (InVitroGen, Paris, France) with random hexamers in a final volume of 20 µL according to the manufacturer's protocols. The quality of the total RNA was assessed by the intensity of 28S and 18S bands after denaturing agarose electrophoresis. The RNA concentration was determined by UV spectrophotometry. One round of cDNA synthesis was performed in a thermal cycler (65°C for 5 minutes before adding RT mix, 42°C for 50 min, and 65°C for 5 minutes).

PCR was performed according to the manufacturer's protocol (0.25 µmol/l of primers from SABiosciences; OPRM1, RefSeq Accession NM_000914.2, band size: 85 bp) and 3 mmol/l of MgCl2, 1 µL of DMSO 20%, 2 µL of PCR mix containing Taq polymerase) in a 20 µL final volume with a thermocycler (LightCycler, Roche, France). The HotStart Taq polymerase (Roche Diagnostics, Meylan, France) was activated by heating for 8 min at 95°C. Duplicate PCRs were performed for 50 cycles with 20 s of denaturation at 95°C, 20 s of hybridisation at 60°C followed by 20 s of elongation at 72°C for each cycle. The quality of products was confirmed by gel electrophoresis to confirm OPRM1amplification size (85 pb).

### Determination of Morphine Levels in Critically Ill Patients

#### Ethics statement

This part of the study was approved by our institutional review board for human experimentation (Study n°3/98 of the 09/12/2003 from the “CPPRB d'Alsace n°1 Strasbourg”). Written informed consent was obtained from each participant or from an authorized representative before enrollment. Patients admitted between July and September 2007 to our intensive care unit (ICU) were prospectively considered for inclusion. Samples from healthy volunteers served as controls. Patients that were given exogenous morphine or codeine before or during admission were excluded. After centrifugation, serum was collected in tubes without anticoagulant agents in order to prevent interactions with endogenous alkaloids.

Patients (n = 48) were divided into five groups according to ASCCP/SCCM (American College of Chest Physicians/Society of Critical Care Medicine [40]; Table 1) criteria: (*i*) healthy donors, (*ii*) patients with Systemic Inflammatory Response Syndrome (SIRS), (*iii*) patients with sepsis (S), (*iv*) patients with severe sepsis (SS), and (*v*) patients with septic shock (SSH). Body temperature, positive microbiological results, white cell count and inflammatory markers (C-reactive protein, procalcitonin) were documented.

**Table 1:** Clinical characteristics of patients.

	SIRS					Sepsis					Severe Sepsis					Sept_ic_ Shock				
	*n*	*MEAN±SD*	*CI*	*X0.5*	*IQR*	*n*	*MEAN±SD*	*CI*	*X0.5*	*IQR*	*n*	*MEAN±SD*	*CI*	*X0.5*	*IQR*	*n*	*MEAN±SD*	*CI*	*X0.5*	*IQR*
Age [years]	13	64.8±14.4	[56,1; 73,6]	66	[52; 77]	11	70.3±11.6	[62,5; 78]	69	[67; 79]	8	66±17.5	[51,4; 80,6]	70	[52,5; 80]	10	71±5.5	[67; 75]	70	[66,8; 77,2]
Length of stay [days]	13	13.2±11	[6,5; 19,8]	7	[5,5; 19,5]	11	9.5±8.9	[3,6; 15,5]	7	[5; 9]	8	9.6±8.3	[2,7; 16,5]	6.5	[5; 13,2]	10	24.3±32	[1,4; 47,2]	13.5	[4; 30,2]
Procalcitonin [μg/L]	13	5±9.2	[−0,6; 10,5]	0.2	[0,1; 6,4]	11	5.8±13.2	[−3,1; 14,7]	0.5	[0,1; 5,7]	8	6.2±12.6	[−4,3; 16,7]	1.7	[0,2; 5,6]	10	64.1±126	[−26,1; 154,3]	11.2	[1,4; 63,6]
WCC [G/L]	13	13100±4005	[10679,8; 15520,2]	14900	[10050; 16300]	11	12855±4652	[9729,5; 15979,6]	11600	[10000; 13800]	8	12225±2674	[9989,9; 14460,1]	12000	[10125; 13675]	10	14780±8218	[8901; 20659]	13600	[10100; 18350]
CRP [mg/L]	13	68.6±74.1	[23,8; 113,4]	24.6	[7,8; 133,7]	11	102±116.3	[23,8; 180,2]	63	[29,5; 114]	8	154.2±154.7	[24,8; 283,5]	119.4	[19,9; 303]	10	144.3±103.5	[70,3; 218,3]	115.7	[86,9; 182,2]
Creatinin [μmol/L]	13	132.4±65.4	[92,9; 171,9]	121	[94,5; 158]	11	118.3±48.4	[85,7; 150,8]	110	[81; 147]	8	155.6±59.2	[106,1; 205,1]	140.5	[109,5; 206,2]	10	208.9±159.5	[94,8; 323]	158	[86; 280,2]
SAPS	13	51.9±20.4	[39,6; 64,2]	53	[33,5; 70,5]	11	41±8.1	[35,6; 46,4]	39	[36; 50]	8	52.5±27	[29,9; 75,1]	46	[34,5; 67]	10	61±19.7	[46,9; 75,1]	55.5	[42,5; 78,8]
LODS	9	4.9±2.5	[3; 6,8]	5	[2; 7,5]	10	3.3±1.6	[2,1; 4,5]	3.5	[2; 5]	8	6.9±5.4	[2,4; 11,4]	5	[2,2; 10,8]	9	6.1±4.3	[2,8; 9,4]	5	[3; 10,5]
SOFA	9	6.7±4.1	[3,5; 9,8]	7	[3,5; 9,5]	10	3.7±2.3	[2; 5,4]	3	[2; 6]	8	7.1±4.4	[3,5; 10,8]	6	[3,2; 11]	9	7.6±2.8	[5,4; 9,7]	8	[4,5; 9,5]
ODIN	13	2.6±1.1	[1,9; 3,3]	3	[2; 3]	11	1.6±1.5	[0,6; 2,6]	2	[0; 3]	8	3±1.1	[2,1; 3,9]	3	[2; 3,8]	10	3.3±0.9	[2,6; 4]	3	[2,8; 4]

SIRS: Systemic Inflammatory Response Syndrome; CRP = C-reactive protein; WCC = White Cell Count; SAPS = Simplified Acute Physiology Score; LODS = Logistic Organ Dysfunction System; SOFA = Sepsis-related Organ Failure Assessment; ODIN = Organ Dysfunction and/or Infection; SD = standard deviation, CI = confidence interval; IQR = interquartile range; X_0.5_ = Median.

* = p<0,05 compared to SIRS.

Daily serum morphine analysis (20 µl) was performed during the first 3 days after admission. Routine physiological and biochemical variables were recorded and Simplified Acute Physiological Scores (SAPS) were calculated according to standards [40].

### Statistical Analysis

R version 2.80 and MINITAB V15 (Minitab Inc., France) were used for data analysis. P levels <0.05 were considered significant. Data failed to be normally distributed (Q-Q plots and Shapiro-Wilk test), so that non-parametric analysis was performed as described below.

#### Statistical analysis of neutrophil secretion experiments

In order to assess morphine and IL-8 secretion after stimulation with IL-8 or LPS, six or seven independent experiments were performed. Mann-Whitney test was performed to test for significant changes. For multiple comparisons, the significance level was adjusted using Bonferroni correction.

#### Statistical analysis of μ opioid receptor expression experiments

Statistical analysis was performed using Friedman's test followed by Wilcoxon-Wilcox procedure, allowing multiple comparisons between groups.

#### Statistical analysis of morphine levels in critically ill patients

Kruskal-Wallis test followed by Dunn's procedure for post-hoc analysis was performed for data which were not normally distributed.

## Results

### Characterization of Endogenous Morphine Present in Neutrophils

Four different batches of neutrophils were extracted (140 to 200.10^6^ cells per extract) and purified. The fraction resulting from the elution procedure was analyzed with a Q-TOF MS-MS approach. Comparison with a morphine standard allowed the unambiguous identification of morphine (m/z = 285.9 Da) and its degradation fragments in the 4 neutrophil extracts (data not shown). No M3G, M6G was detected. The amount of the morphine determined by ELISA and MS analysis was 0.32+/−0.2 pg/million of neutrophils ± SD (n = 4). This value is lower than the value previously found (12.33±5.64 pg/million cells ± SD) [19], however, we could confirm that morphine is present in human neutrophils

### Subcellular Localization of Morphine in Human Neutrophils

First, the specificity of the antibody 6D6 was tested using a competitive ELISA (Fig. 1A). It appears that the 6D6 antibody displays a high affinity for M6G (50% displacement point = 0.07 nM or 0.038 ng/ml), but also reacts with morphine (18.82 nM or 3.91 ng/ml), M3G (13 nM or 4.98 ng/ml) and codeine (7.95 nM or 2.29 ng/ml). According to our MS-MS analysis, only morphine is produced by neutrophils, indicating that the immunolabel found with the 6D6 antibody only reflects presence of endogenous morphine.

Second, using laser confocal microcopy, the labeling obtained with the anti-morphine mouse monoclonal antibody was compared to that of lactoferrin, a specific marker of secondary granules (or specific granules) present in neutrophils [41]. Morphine immunolabeling showed a punctate pattern in the cytoplasm, similar to that obtained with an anti-lactoferrin antibody (Fig. 1B, upper panel). Well-defined restricted immunolabelings in specific structures were observed using higher magnification (Fig. 1B, lower panel). Superimposition of the two labelings revealed a colocalization within lactoferrin-containing granules (*i.e*., secondary granules; Fig. 1B, yellow label).

Finally, the presence of morphine in erythrocytes which was described in previous reports [20] was studied by immunocytochemistry. Fresh blood drops were spread on a glass slice and immunocytochemistry using the 6D6 antibody was performed. Fig. 1C shows the presence of morphine-like immunoreactivity in neutrophils, whereas no immunoractivity is present in erythrocytes.

Control experiments established the specificity of the labeling in granules using either secondary antibodies alone or the mouse anti-morphine antibody blocked with morphine prior to its use in immunocytochemistry experiments (Fig. 1D).

In conclusion, this experiment revealed morphine immunoreactivity within the secondary granules of human neutrophils, suggesting its potential secretion upon cell stimulation.

### Morphine Secretion from Human PMN

The amount of morphine released into the medium by neutrophils after LPS or IL-8 stimulation was detected with a sensitive morphine-specific ELISA kit (Fig. 2A). Control experiments using IL-8 and cytochalasin B or LPS in the absence of neutrophils ruled out cross-reactivity of these substances with the ELISA (data not shown). Figure 2B (upper panel) shows the increase of morphine secretion in cultured human neutrophils stimulated with LPS (158±35 pg/10^6^ neutrophils ±SD, n = 6). A low basal level of morphine secretion was observed in the absence of LPS (10±24 pg/10^6^ neutrophils ±SD, n = 6). Secretion efficiency was checked by Western blot analysis (50 µg) using an antibody against lactoferrin which is present in and secreted by secondary granules [41]. Lactoferrin was detected in both non stimulated and LPS stimulated samples. However, the amount of lactoferrin was higher in the supernatant of LPS stimulated neutrophils (Fig. 2B, lower panel). The presence of lactoferrin in the supernatant of unstimulated neutrophils might be caused by the basal secretion from neutrophils activated by their artificial environment, however, the rate of cell death was not increased compared to controls.

In order to observe whether morphine secretion is Ca^2+^-dependent, identical experiments were performed in the absence of Ca^2+^. Without Ca^2+^ in the media, basal secretion remained unchanged (9±21 pg/10^6^ neutrophils ±SD, n = 6), while LPS-induced secretion of morphine decreased significantly (69±15 pg/10^6^ neutrophils ±SD, n = 6, p<0.01).

In parallel, PMN were treated with 50 nM IL-8 for 7 min (Fig. 2C, upper panel) to trigger direct secretion of secondary granules [34], [41]. In the presence of IL-8, morphine secretion was attenuated, but still significant (60±7 pg/10^6^ neutrophils ±SD, n = 6) compared to baseline (16±7 pg/10^6^ neutrophils ±SD, n = 6). Western blot analysis confirmed the presence of lactoferrin in IL-8 treated cell medium (Fig. 2C, lower panel) and its absence in nonstimulated cell medium after 7 min. Without Ca^2+^, the basal secretion of morphine was not significantly changed compared to controls with Ca^2+^ (14±6 pg/10^6^ neutrophils ±SD, n = 6), however, IL-8-induced secretion of morphine decreased significantly under Ca^2+^-free conditions (15±6 pg/10^6^ neutrophils ±SD, n = 6, p<0.01).

In conclusion, the present results demonstrate that human neutrophils secrete morphine *via* a Ca^2+^-dependent mechanism in response to LPS or IL-8 stimulation.

### Effects of Morphine on IL-8 Secretion

**Figure 3:**
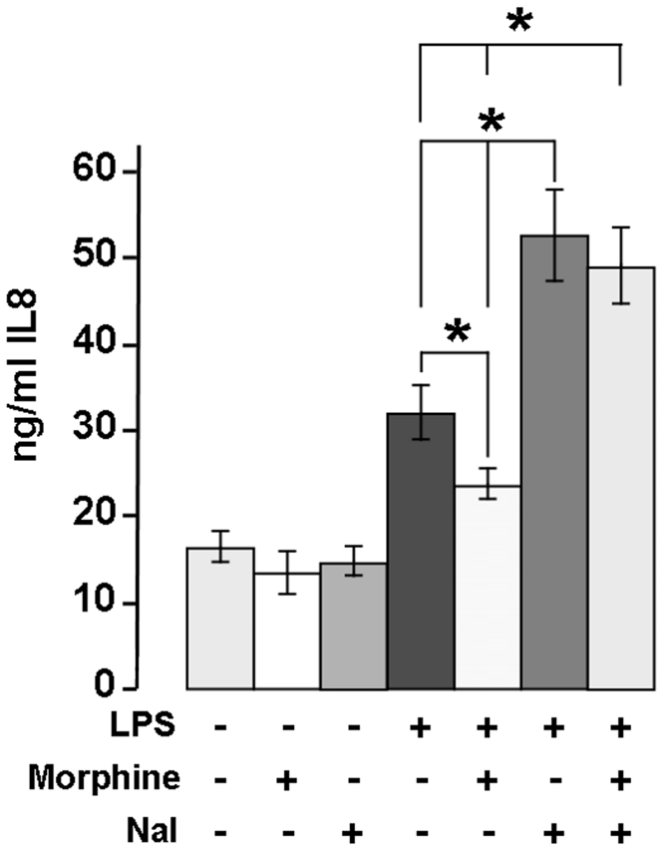
IL-8 secretion from human primary neutrophils. IL-8 secreted from neutrophils expressed as ng/ml of morphine. Morphine was quantified in culture medium after stimulating 1.10^6^ neutrophils with or without LPS (10 ng/ml, 24 h; n = 6), morphine 8 nM (2.5 ng/ml) and naloxone (10 µM). Basal secretion levels were obtained from neutrophils incubated without LPS (n = 6). *: p<0.05 with a Mann-Whitney test.

Human neutrophils (1.10^6^ cells) were stimulated with 10 ng LPS and/or morphine (8 nM corresponding to 2.25 ng/ml) and/or 10 µM naloxone (MOR antagonist) to determine the impact of low morphine concentrations on IL-8 secretion after 24 h (Fig. 3). Using a commercial IL-8 ELISA, no significant differences were found between controls without LPS (no stimulation, 16±2 ng/10^6^ neutrophils ±SD, n = 6), morphine (13±3 ng/10^6^ neutrophils ±SD, n = 6, p>0.05) and naloxone alone (14±1.4 ng/10^6^ neutrophils ±SD, n = 6, p>0.05). Compared to controls LPS significantly increased the release of IL-8 (32±3 ng/10^6^ neutrophils ±SD, n = 6, p<0,05). Costimulation of neutrophils with LPS and morphine decreased the release of IL-8 significantly (24±2 ng/10^6^ neutrophils ±SD, n = 6, p<0,05). Coincubation of neutrophils with LPS and naloxone increased the secretion of IL-8 significantly (53±6 ng/10^6^ neutrophils ±SD, n = 6, p<0,05) compared to LPS-alone (Fig. 3), suggesting a negative feedback mechanism of endogenous morphine secreted upon LPS stimulation. Finally, naloxone was able to significantly inhibit the effect of exogenous morphine on neutrophils treated with LPS compared to LPS and morphine incubation alone (49±5 ng/10^6^ neutrophils ±SD, n = 6, p<0,05).

In conclusion, our results indicate that low concentrations of morphine as found in the serum of patients with sepsis significantly decrease the secretion of IL-8 from neutrophils.

### μ Opioid Receptor Expression on Neutrophils

**Figure 4:**
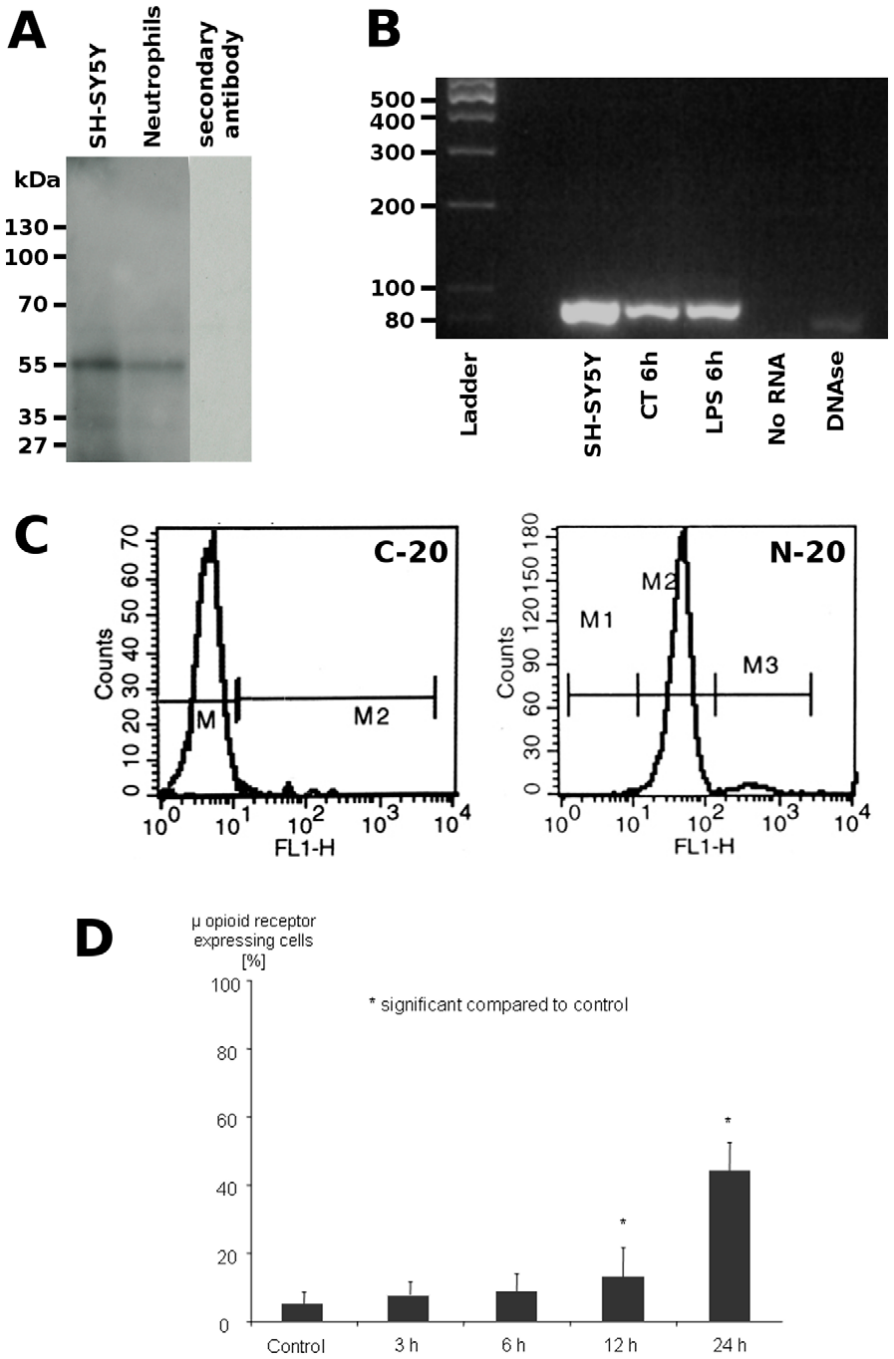
Detection of μ opioid receptor positive cells by and Western Blot, PCR, and flow cytometry. ***A.*** Western blot analysis of 50 µg of SH-SY5Y (positive control) and human neutrophil extracts with the anti-μ opioid receptor (N-20). Control experiment shows the absence of cross reactivity for the secondary antibody. ***B.*** PCR amplification of MOR1 RNA performed on both naïve and LPS treated neutrophil total RNA extracts indicates the presence of a single band at 85 bp. Total RNA from SH-SY5Y cells is used as positive control, whereas negative control were done in absence of RNA and neutrophil RNA extract treated with DNAse. ***C.*** Flow cytometry with live gating on neutrophils using either a C-terminal antibody (C-20) or a N-terminal antibody (N-20). M1, M2 and M3 denote the different histogram gates used for determination of the median of fluorescence intensity ***D.*** *Flow cytometry.* Whole blood was stimulated with LPS (100 ng/ml) for different time intervals followed by labeling of μ opioid receptor expression on neutrophils with FITC-anti-μ opioid receptor (N-20). The percentage of cells showing an increased expression of μ opioid receptors is shown.

Flow cytometry experiments were performed to determine if morphine affects μ opioid receptor expression. An antibody (MOR1 N-20) directed against the N-terminal part of the receptor was used. In order to validate the use of this antibody, we first performed a Western blot analysis of SH-SY5Y (positive control) and human neutrophil cell extracts. In both extracts, immunoreactivity was observed as a band at 55 kDa which is consistent with the expected molecular weight of the μ opioid receptor (Fig. 4A). A control experiment using the secondary antibody alone showed that no cross reactivity exists (Fig. 4A). PCR was used to determine whether MOR1 is expressed in human neutrophil under basal condition and after LPS treatment for 6 h (100 ng/ml). As shown in Figure 4B, amplification of MOR1 RNA performed on both naïve and LPS treated neutrophil total RNA extracts indicates the presence of a single band at 85 bp, which is also present in total RNA from SH-SY5Y cells used as a positive control [10]. This band is absent when RNA is omitted or when the neutrophil RNA extract is treated with DNAse (Fig. 4B).

Incubation of whole blood with the C-terminal antibody C-20 revealed the same median of fluorescence intensity as incubation with FITC-anti-IgG as a negative control (Fig. 4C), indicating that the C-terminal antibody failed to detect μ opioid receptor expression on the neutrophil surface. In contrast, a significantly higher signal was detected with N-20, which is directed against the N-terminus of the μ opioid receptor (Figure 4C) and confirms the results of the Western blot.

Flow cytometry experiments using the N-20 antibody were able to show that, without stimulation, only 5.37±3.34% of neutrophils showed a high level of μ opioid receptor expression. However, after 12 and 24 h stimulation with LPS, this percentage increased significantly (p = 0,031) to 13.28±8.47% after 12 h and 44.4±8.06% after 24 h of LPS stimulation (Fig. 4D). There was no significant change in the median of channel fluorescence intensity in any of the histogram gates used.

Taken together, these results demonstrate that μ opioid receptor expression on neutrophils is significantly increased after stimulation with LPS.

### Characterization of Morphine in the Serum of Patients with Sepsis

**Figure 5:**
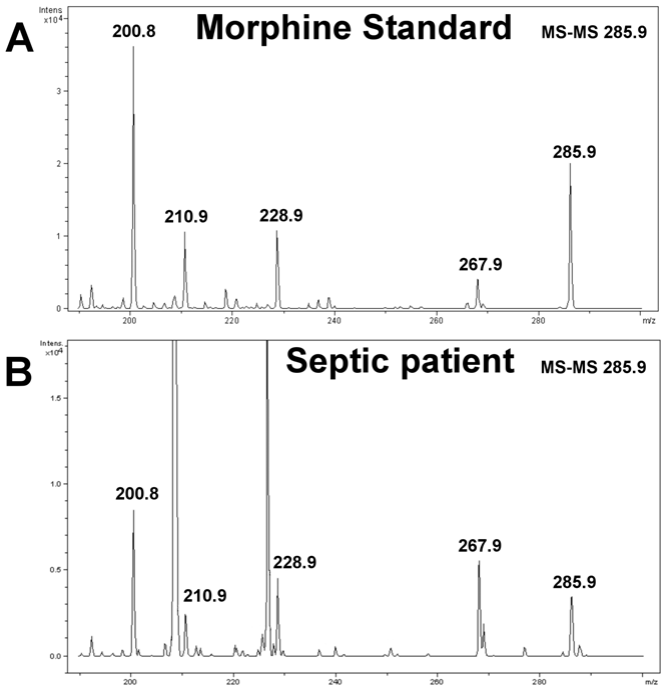
Characterization of morphine in serum of patients with sepsis. *Top*, Q-TOF MS-MS analysis of morphine standard (286.17 Da; 1 pmol). *Bottom*, Q-TOF MS analysis of 1/50 of the morphine present in 10 ml of serum from a patient with sepsis.

Deproteinized serum extracts (3 patients with sepsis and 3 healthy donors) were purified by solid phase extraction. The fraction (1/50) resulting from the elution procedure was analyzed with a Q-TOF MS-MS approach. Comparison with a morphine standard (Fig. 5A) allowed the unambiguous identification of morphine (m/z = 285.9 Da) and its degradation fragments in patient extracts (Fig. 5B). Parallel experiments performed on the serum of healthy donors failed to detect endogenous morphine (n = 3; data not shown).

### Serum Morphine Levels in Critically Ill Patients

Using a morphine-specific ELISA kit, morphine concentrations were quantified in the serum of different patient groups (see Table 1 for patient categorization) with or without infection. The mortality was 30%. In septic patients (sepsis, S, n = 13; severe sepsis, SS, n = 8; septic shock, SSH, n = 10), sources of infection were respiratory, urinary, or digestive (n = 17, 8, and 6, respectively). Gram negative bacteria were involved in 55% of the cases and Gram positive bacteria in 45%. Sedation was carried out without opiates using midazolam and propofol according to standard protocols. There was no difference between the groups with regard to mean length of stay on the intensive care unit (ICU), age and sex. As expected, SOFA scores were higher in patients with severe sepsis and septic shock (p = 0.041).

**Figure 6:**
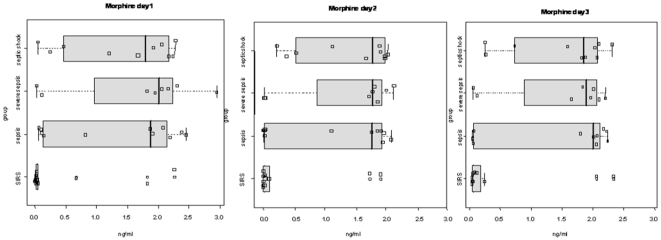
Three-day follow up of morphine concentrations in infected and uninfected patients. Boxplots of endogenous morphine concentrations measured in the serum of patients with sepsis (n = 13), severe sepsis (n = 8) and septic shock (n = 10), compared to critically ill patients with SIRS (n = 6) over 3 days of monitoring. Patients with sepsis, severe sepsis and septic shock had significantly higher serum concentrations of morphine than patients with SIRS (Sepsis vs. SIRS, p<0.05; severe sepsis vs SIRS, <0.01; septic shock vs SIRS, p<0.01).

ELISA analysis revealed that morphine concentrations were significantly higher in the serum of septic patients compared to patients with SIRS during the three days of monitoring (Fig. 6). However, no significant difference was observed between patients with sepsis, severe sepsis and septic shock. No detectable amount of morphine was found in 6 healthy controls (data not shown); and CRP, white cell count and procalcitonin were within normal range in these individuals (n = 6; data not shown). The interquartile ranges for morphine concentrations in patients with sepsis, severe sepsis or septic shock were [0,011; 2,194], [0,542; 2,273] and [0,417; 2,191], respectively. This correlates well with the morphine concentration (2.25 ng/ml) used for our *in vitro* experiments to evaluate the influence of morphine on IL-8 secretion. Thus, it seems likely that the morphine levels found in septic patients may play a role in regulating neutrophil IL-8 secretion.

In conclusion, our data indicate unambiguously that serum morphine concentrations increase specifically in patients with life-threatening infection.

## Discussion

In the present study, we have demonstrated (*i*) that morphine is present in human neutrophils and is colocalized with lactoferrin, an antimicrobial peptide released during infection [42] from the secondary granules of neutrophils, (*ii*) that LPS and IL-8 trigger the Ca^2+^-dependent secretion of morphine and lactoferrin *in vitro*, (*iii*) that endogenous morphine concentrations are significantly increased in the serum of patients with septic conditions, (*iv*) that the concentration of morphine found in sepsis inhibits IL-8 release from neutrophils and finally (*v*) that μ opioid receptor surface expression is increased after stimulation of white cells with LPS.

Our findings provide further evidence for the role of endogenous morphine [4] as a signaling molecule that can act through μ opioid receptors expressed on immune cells. The activation of the morphinergic system as demonstrated by an increase in morphine secretion as well as μ opioid receptor expression by neutrophils, underlines the hypothesis that endogenous morphine is part of the innate immune response to stress, and in particular, infection. In addition, our data suggest a new link between endogenous morphine expression and host defense against infection.

During gram-negative sepsis bacterial LPS leads to the activation of PMN and triggers the synthesis and secretion of proteases, cytokines (*e.g*., IL-6 and IL-8), and toxic radicals from immune cells [43], [44]. Among these cytokines, IL-8 acts as a potent stimulator of PMN and represents a major chemoattractant for these cells [45], [46]. PMN participate in the host defense *via* the secretion of particular antimicrobial peptides including lactoferrin, which is present in secondary granules and is released upon LPS stimulation [42], [47]. Despite their important role in destroying the pathogen, compounds released by PMN inflict damage to host cells [48] and contribute to organ dysfunction during systemic infections. Thus, regulatory mechanisms including the hypothalamic–pituitary–adrenal axis [49], as well as PMN autoregulatory mechanisms such as apoptosis [50], are necessary to prevent local tissue destruction [51]. Recently, the μ opioid receptor agonist DAGO in concentrations as low as 10 nM was shown to significantly decrease the secretion of IL-8 from neutrophils after LPS stimulation [38]. We now demonstrate that LPS and IL-8 trigger the secretion of morphine from human PMN *in vitro*. Our results also indicate that low concentrations of morphine (8 nM coresponding to 2.25 ng/ml) consistent with the amount of morphine found in the serum of septic patients, significantly decrease the secretion of IL-8 from human neutrophils and that this inhibition can be reversed by naloxone. Therefore, it is tempting to speculate that morphine present in the serum of septic patients is secreted from neutrophils and is involved in the regulation of immunocyte activity. Interestingly, a recent paper reported that concentrations of morphine as low as 10–100 nM enhanced migration of primary microglial cells toward adenosine diphosphate. This effect was reversed by naloxone, as well as CTAP, indicating μ opioid receptor involvement [24]. These data strongly suggest that release of endogenous morphine may affect immune cells either directly or *via* central regulatory mechanisms.

The presence of morphine in human PMN and mononuclear cells is a matter of dispute: Whilst the presence of morphine was described by Zhu et al [19], Boettcher and colleagues [20] failed to demonstrate morphine production within neutrophils. In the present study, we have unambiguously demonstrated the presence of morphine in PMN, however, the amount of morphine found was well below the concentration (0.32+/−0.2 pg/million PMN ± SD *vs* 12.33±5.64 pg/million cells ± SEM) described before [19]. A very low amount of morphine was previously found in erythrocytes by Boettcher and colleagues [20], however, we failed to show the presence of morphine-like immunoreactivity in these cells in our experiments.

Atypical cholinergic regulation of morphine secretion from human white blood cells has been demonstrated before [22]. Our present study shows that morphine is present in secondary granules and is secreted from neutrophils in response to LPS and IL-8 exposure, suggesting that at least part of the circulating morphine in the blood of septic patients stems from PMN. Interestingly, this is supported by the observation that LPS administration in animals dramatically increases the amount of circulating [29] and cerebral endogenous morphine [28]. However, the low amount of morphine produced and secreted by neutrophils seems not sufficient to reach the morphine concentrations observed during sepsis. Thus, during systemic infection morphine release from the adrenal gland [4], [11], [15], [36], its delayed glucoronidation in the liver [12] and morphine synthesis in the nervous system [3], [28], [52] may potentially contribute to elevated morphine levels in the systemic circulation.

Morphine preferentially binds to μ opioid receptors, a large family of seven-transmembrane receptors derived from a single OPRM1 (opioid receptor μ) gene. However, endogenous opioid peptides such as β-endorphine, endomorphines and enkephalins are also able to bind to μ opioid receptors. Serum levels of endogenous opioid peptides are elevated in response to stress and also in septic patients [53], [54]. Treatment with opiate antagonists for the cardiovascular effects of septic shock was proposed more than 20 years ago [55], indicating a key role of opioid peptides in the stress response. It is likely that part of this response is mediated *via* opioid receptors expressed in different tissues including endothelia, leukocytes and myocardium. Endogenous opioid peptides present in the serum of septic patients may compete with morphine for μ opiate receptor binding. However, no effects of these interactions have been described yet in septic patients. Thus, further studies are warranted to investigate how endogenous opioid peptides may influence or possibly regulate morphine-induced neutrophil inhibition in response to stress and infection.

Stimulation of μ opioid receptors (for review: [56], [57]), triggers various effects including direct inhibition of immunocyte function. Human granulocytes and monocytes express μ opioid-like receptors as demonstrated by specific and saturable binding of dihydromorphine [58]. μ opioid receptors were also found on human and monkey lymphocytes [59]. A specific μ opioid receptor isoform, the μ3 receptor (morphine selective but opioid peptide insensitive) has also been characterized in human monocytes and granulocytes as well as in endothelial cells [16], [60]. The existence of numerous splicing variants underpins the complexity of the morphinergic system [57], [60], [61]. To date, fourteen human μ opioid receptor isoforms have been identified [56], [57], [62] with various affinities for morphine and its derivatives (Ki in the nanomolar range with low affinity for the recently described isoform mMOR-1B4 [56], [57]), suggesting that different morphine concentrations are needed for specific receptor isoform activation. In addition, homo- or hetero-dimerisation of μ opioid receptor subtypes was recently found to occur and to induce switch signaling [63].

Although it has been shown before that LPS increases μ opioid receptor expression in the rat mesenteries [64], to our knowledge our results demonstrate for the first time that μ opioid receptor surface expression on neutrophils is increased after stimulation with LPS. The expression of μ opioid receptors is well documented for immune and endothelial cells [60], [65], [66]. Amongst other opioid receptor subtypes, PMN express the μ3 opioid receptor subtype coupled to constitutive nitric oxide (NO) release [60], [67], [68], [69]. Due to its immunoinhibitory nature [70], [71], it has been postulated that endogenous morphine down-regulates immune function to maintain a balance between pro- and anti-inflammatory processes. In this regard, morphine is known to dramatically affect innate and adaptive immune responses [72], [73], [74], [75]. For example, morphine affects lymphocyte proliferation [76], [77], natural killer T-cell activities [78], [79], antibody production [80], [81], and the number of circulating leukocytes [82]. Inhibition of phagocyte function, such as chemotaxis, phagocytosis, and surface receptor expression after morphine exposure *in vitro* is, in part, a consequence of NO synthesis [69] and involves the modulation of the transcription factors NF-κB and AP-1 [67], [68], [70], [83], [84]. In addition, recent data on rat microglial cells treated with adenosine diphosphate have shown that low concentrations of morphine induce a biphasic effect, with an initial phase involving PI3K/Akt pathway activation that leads to an increase of cell migration [24], followed by a longer-term phase with increased expression of Iba1 and P2X4 receptor protein, leading to a greater cell migration.

Furthermore, different μ opioid receptor splicing variants are expressed by PMN, suggesting that these μ receptor subtypes may mediate neutrophil responses during infection. Interestingly, the μ opioid receptor possesses an affinity for morphine with an IC50 around 10nM [85] compatible with the concentration of morphine observed in the patients with sepsis (8nM or 2.5ng/ml) suggesting that auto- and/or paracrine regulation involving immune cells and possibly the endothelium exists.

Several studies have linked endogenous morphine with inflammatory conditions. Endogenous morphine was found in the plasma of patients after cardiovascular bypass which induces a well-described systemic inflammatory response [4], [26]. Interestingly, our study shows that morphine levels found in SIRS patients (mean between 0,39 and 0.57 ng/ml) were comparable to those described after cardiovascular bypass (mean of 0.75 ng/ml) [26], wheras morphine was not detected in healthy controls.

Sepsis [86], which is a condition associated with a systemic inflammatory response caused by bacterial, fungal or viral infection, is one of the main causes of mortality in intensive care units [87]. The diagnosis of sepsis is based on clinical symptoms [40], although several endogenously produced proteins, such as procalcitonin or C-reactive protein (CRP), have been proposed as markers of infection and are used in clinical practice [88].

Our study demonstrates a significant increase of serum morphine levels in patients with severe sepsis or septic shock, while in critically ill patients suffering from SIRS and controls, morphine levels were significantly lower. No significant difference was observed between patients from the three septic groups, but morphine was undetectable in the serum of healthy donors. Our results indicate that endogenous morphine might represent a new biomarker for the diagnosis of sepsis, however, an extended cohort of patients is needed to validate this hypothesis.

Opioid analgesia is a standard component of sedation in critical care, with synthetic opiates such as fentanyl and its derivatives being most frequently used. The clinical significance of sedation-induced immunosppression is still under debate. So far, no direct effect of fentanyl in clinically relevant concentrations could be demonstrated on neutrophil functions and fentanyl does not bind to opioid receptors expressed on neutrophils [84]. It remains unclear whether the application of exogenous opiates interferes with the signaling of endogenous morphine *via* direct or centrally mediated mechanisms.

Interestingly, our data reveal that concentrations of morphine found in the serum of septic patients are in range with the affinity of μ opioid receptors [56]. This correlation suggests a putative physiological role during sepsis. Based on morphine's immunoinhibitory activity, it is tempting to propose that during sepsis, increased morphine levels may down-regulate immune and vascular tissue responses to prevent organ damage caused by excessive inflammation. However, recent data suggest that low concentrations of morphine (10–100 nM), synergistically with other factors increase immune activity (*i.e.,* migration) [24]. In addition, several studies in rodents have shown that morphine in clinically relevant doses exacerbates inflammation and increases sepsis mortality [89], [90]. Thus, we can not exclude that the increase of endogenous morphine may also act in favor of proinflammatory responses in certain cells. As demonstrated in our study, endogenous morphine concentrations are in the nanomolar range while morphine concentrations after administration of clinically relevant doses for analgosedation in intensive care are 10 to 100-fold higher [91]. It is unclear so far whether the different concentration range of exogenous and endogenous morphine affects neutrophil function in contrasting ways. Morphine levels can be related, in part to PMN secretion, but also to secretion from other organs including adrenal gland and liver. Therefore, direct monitoring of neutrophil function in relation to the plasma morphine concentrations is required to eventually answer this question.

Taken together, our present observations together with previously published data, support the hypothesis that endogenous morphine represents a modulator of immune cell activity. Our data indicate for the first time that endogenous morphine is secreted from human neutrophils after LPS or IL-8 stimulation. In patients with generalized infection such as sepsis, severe sepsis, and septic shock serum levels of endogenous morphine were found to be dramatically increased, whereas inflammatory states without infection such as SIRS did not lead to profound endogenous morphine rises. In addition, our results indicated that low concentrations of morphine inhibit LPS-induced IL-8 secretion by neutrophils. Further investigations, including clinical studies, are required to fully understand the effects of circulating morphine on immune function in sepsis.
